# AV Interval Optimization - A Step Towards Physiological Pacing in Patients with Normal Left Ventricular Function

**Published:** 2010-09-05

**Authors:** Shomu Bohora

**Affiliations:** U.N. Mehta Institute for Cardiology and Research Centre, Civil Hospital Campus, Ahmedabad, India

**Keywords:** AV Interval Optimization, Physiological Pacing

"In wilderness I sense the miracle of life, and behind it our scientific accomplishments fade to trivia."  Charles A. Lindbergh.

Pacemakers have evolved over a period of time trying to mimic the normal response rates, conduction and activation characteristics, though are still far from what nature has bestowed upon us. Better understanding of cardiac physiology and hemodynamics has led to current available pacing technology and we do recognize now that to achieve physiological pacing we should have an appropriate heart rate response, ventriculo-ventricular (VV) synchronization and atrio-ventricular (AV) synchronization.

Patients receiving rate responsive pacemakers for sinus node dysfunction, in spite of using various sensors and rate response algorithms [1-5], still do not truly have an appropriate heart rate response, especially in absence of physical stress. There is a need to develop sensors, based on which an algorithm can be developed to achieve a heart rate response, which truly mimics to what a normal sinus node would behave in response to both physical and mental stress. In patients with heart block who have atrial sensing based ventricular pacing, the heart rate response remains appropriate if the sinus node is normal.

Right ventricular (RV) pacing represents a non-physiological activation of the heart causing wide QRS (left bundle branch block) with electrical and mechanical VV dyssynchrony [5]. Higher percentage of ventricular pacing in patients with intact AV node has been found to be associated with increased incidence of atrial fibrillation and heart failure on follow up [6-10]. Algorithms to prevent ventricular pacing are effective in reducing unnecessary ventricular pacing in patients with normal AV conduction and sick sinus syndrome. However these algorithms cannot be applied to patients with advanced heart block in which there is need for mandatory ventricular pacing. To avoid detrimental effects of VV synchrony alternate site RV pacing [11-15] and biventricular pacing have been described. [16,17] Alternate site pacing studies have shown mixed results [11-15]. Left sided lead placement, non-physiological epicardial pacing and procedure and pacing related complications with the higher overall cost involved in doing biventricular pacing procedure represents a significant limitation for advising it as a routine. VV dyssynchrony possibly would remain a limitation in achieving total physiological pacing till further conclusive evidence of newer pacing methods is demonstrated.

Optimal AV interval at rest ranges from 100 to 150 milliseconds. In normal individuals the AV interval shortens with increased heart rate during exercise in a predictable and linear fashion. Most pacemakers have a programmable shortening of AV delay at higher rates, the hemodynamic benefits of which have not yet been shown [1]. The aim of optimizing AV delay in patients with heart failure is to increase diastolic filling and at the same time maintain biventricular pacing so as to maximize cardiac output. In patients with heart failure and LV dysfunction even a small improvement in cardiac output, as obtained by optimizing AV delay, may result in significant clinical improvement. AV optimization is routinely done using echocardiographic techniques of which Ritter's method is the most commonly used [18]. Device based algorithms like QuickOpt is also available and is currently being evaluated for its effectiveness in comparison to echocardiographic methods [19]. Optimizing AV synchrony and hence AV delay is routinely not advised in patients receiving pacemakers without heart failure.

An electrocardiogram based method to determine optimal AV interval is described by Sorajja et al [20] in this issue of the journal, in which P wave duration correlates with a correction factor of 1.26 with an optimal AV interval, as determined by Ritter's method of AV optimization on echocardiography. Such simple technique can be used for effectively programming optimal AV delay routinely once validation by large trials occur, so as to achieve better hemodynamics without the need for time consuming echocardiographic techniques or till the time echocardiographic optimization is routinely planned. This study, though with its limitations of having a small cohort of elderly patients and optimization evaluated only at rest, presents an attractive alternative to echocardiography based techniques to calculate and program optimal AV delay.

Based on echocardiographic parameters and natriuretic peptide levels, AV delay optimization is found to be beneficial in patients with normal LV function in short term small studies [21-25]. There exists hardly any long term study to demonstrate benefits of routine optimization of AV delay in patients having normal LV function and receiving pacemakers for heart block. Hence it would be difficult to justify echocardiography based AV optimization in all such patients. However it seems appropriate to aim to program an optimal AV delay in all patients receiving pacemakers, based on data from heart failure patients and short term studies. Can the findings of this study be extrapolated for use in AV optimization in patients treated with devices for heart failure? Larger studies in patients with and without LV dysfunction and heart failure would be required to validate the results of this pilot study for incorporating it in clinical practice to achieve better long term outcomes. We still have a long way to go before we can mimic with pacemakers the normal electrical activity of the heart.

Adopt the pace of nature:  her secret is patience  - Ralph Waldo Emerson

Look deep into nature, and then you will understand everything better  - Albert Einstein
